# Mortality Patterns in the West Bank, Palestinian Territories, 1999-2003

**Published:** 2008-09-15

**Authors:** Niveen ME Abu-Rmeileh, Abdullatif Husseini, Rita Giacaman, Omar Abu-Arqoub., Mutasem Hamad

**Affiliations:** Institute of Community and Public Health, Birzeit University; Institute of Community and Public Health, Birzeit University, Ramallah, West Bank, Palestinian Territory; Institute of Community and Public Health, Birzeit University, Ramallah, West Bank, Palestinian Territory; Palestinian Ministry of Health, Palestinian Health Information Center, Nablus, Palestinian Territory; Palestinian Ministry of Health, Palestinian Health Information Center, Nablus, Palestinian Territory

## Abstract

**Introduction:**

The West Bank in the Palestinian Territories is undergoing an epidemiologic transition. We provide a general description of mortality from all causes, focusing on chronic disease mortality in adults.

**Methods:**

Mortality data analyzed for our study were obtained from the Palestinian Ministry of Health in the West Bank for 1999 through 2003. Individual information was obtained from death notification forms.

**Results:**

A total of 27,065 deaths were reported for 1999 through 2003 in the West Bank, Palestinian Territories. Circulatory diseases were the main cause of death (45%), followed by cancer (10%) and unintentional injuries (7%). Among men, the highest age-standardized mortality rates (ASMRs) were due to diseases of the circulatory system, cancer, and unintentional injuries. Among women, the highest ASMRs were due to circulatory disease, cancer, and diabetes mellitus. Of the circulatory diseases, the highest ASMRs for men were due to acute myocardial infarction and cerebrovascular disease. ASMRs attributable to circulatory system diseases were similar for women. Lung cancer was the largest cause of cancer mortality for men; breast cancer was the largest cause for women.

**Conclusion:**

Because of the high mortality rates, the risk factors associated with chronic diseases in the Palestinian Territories must be ascertained. Medical and public health policies and interventions need to be reassessed, giving due attention to this rise in modern-day diseases in this area.

## Introduction

The West Bank, Palestinian Territories, is undergoing a transition characterized by rapid urbanization (1) and changing lifestyles. According to the Palestinian Central Bureau of Statistics (PCBS), approximately 40% of West Bank residents lived in rural areas in 2006 (1,2), compared with 62% in the early 1990s (3). At the same time, the Palestinian Territories have been undergoing an epidemiologic transition characterized by a persistent burden of infectious diseases typical of developing countries and a rise in noncommunicable (chronic) diseases such as cardiovascular disease, hypertension, diabetes mellitus, and cancer. High prevalences of type 2 diabetes and obesity were observed in urban and rural Palestinian areas beginning in the late 1990s, and the rates are rising (4,5).

In 2001, the West Bank had a population of approximately 2.1 million (6). Since 2000, the Palestinian population has endured intense conflict characterized by severe restrictions on the movement of Palestinian people and goods, difficulties of access to health services, and spiraling poverty, which negatively affect living conditions and health status (7,8).

Medical and public health providers shifted focus because of these conditions, and they continue to focus on coping with the emergency situation in the country. The Ministry of Health of the Palestinian Authority has a reliable surveillance system for communicable disease, aiming to prevent outbreaks. However, until recently, vital registration and reliable information on causes of death were unavailable. Data on causes of death were registered at the Israeli civil administration until 1994; after the 1993 Oslo Accords, information on causes of death was not transferred to the Palestinian Ministry of Health. Palestinians began to collect information on causes of death in 1994.

The improvement in surveillance and vital registration systems brought about by emergency conditions since 2000 offers us the opportunity for the first time to describe mortality from all causes in the West Bank, focusing on mortality from chronic diseases in adults, and to raise awareness of what has been an unrecognized public health problem.

## Methods

Data on causes of death were obtained from the Palestinian Health Information Centre (PHIC) in the West Bank from 1999 through 2003. There are 2 centers for PHIC: 1 is in Nablus, which is in the center of the West Bank, covering registration for the entire West Bank; the other is in Gaza City, Gaza Strip. PHIC personnel obtain copies of death notifications from the local health directorates, which issue burial permits and death certificates and keep the Ministry of Health's copy of death notifications. Death certificates are completed by physicians. The notification includes the full name of the deceased, address, religion and ethnicity, marital status, date of birth and date of death, place of death, occupation, direct and underlying cause of death, and information about the person who reported the event. The direct and underlying causes of death are checked and coded by a medical committee appointed by the Ministry of Health. The data included in this analysis are based on the underlying cause of death and coded according to the *International Statistical Classification of Diseases and Related Health Problems, 10th Revision* (ICD-10) (9).

According to the World Health Organization (WHO) criteria, the Palestinian mortality data are classified of medium quality (10). The quality of data is judged by the proportion of deaths assigned to the codes for "symptoms, signs, and abnormal clinical and laboratory findings, not elsewhere classified" (ICD-10 codes R00-R99), "event of undetermined intent" (Y10-Y34 and Y872), cardiac arrest, heart failure, and secondary or unspecified cancer sites. For the Palestinian data, the percentage of ill-defined conditions ranged from 17% to 22%. The completeness of data at the West Bank level ranged from 72% to 80% (11).

Causes of death were recoded into 5 major groups: 1) communicable diseases, which includes infectious and parasitic diseases (A00-B83), blood diseases and immune disorders (D50-D64), respiratory infections (J00-J99), perinatal conditions (P00-P96), nutritional deficiency (E40-E64), and maternal conditions (O00-O99); 2) cancers, including all malignancies (C00-C95); 3) diabetes mellitus (E10-E14); 4) circulatory diseases including hypertension (I10-I15), acute myocardial infarction (AMI) (I21-I23), chronic ischemic heart disease (I25), heart failure (I50), cerebrovascular disease (I60-I69), rheumatic heart disease (I05-I09), pulmonary embolism (I26-I28), and cardiomyopathy (I42); and 5) unintentional injuries. The unintentional injuries group includes all causes of injuries (S00-X39) except suicide and intentional self-harm (X60-X84), causes of death that were grouped under intentional injuries for this analysis.

**Figure 1:**
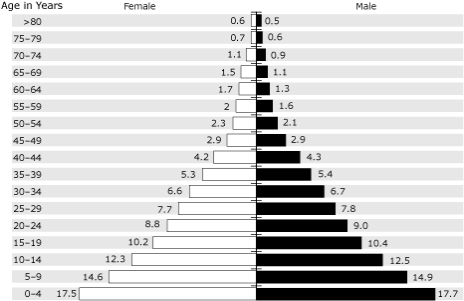
Palestinian Territories Population Structure, 2000. Adapted from reference 12.

ASMRs were calculated using the Palestinian population structure for the year 2000 (Figure 1) and then were standardized to the world population structure.

**Table:** 

## Results

**Figure 2:**
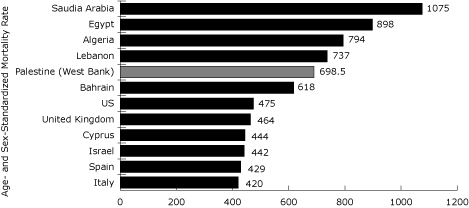
Age- and Sex-Standardized Mortality Rate per 100,000 Population for Selected Countries, 1999-2003.

A total of 27,065 deaths were recorded in the death registry in the West Bank from 1999 through 2003. The ASMR for the West Bank was high (689.5 per 100,000 population per year; 95% confidence interval [CI]: 681.3-697.7 per 100,000 population per year) compared with that of other Mediterranean countries such as Italy and Spain. However, the ASMR was lower than that of other Arab countries such as Egypt and Saudi Arabia (Figure 2).

**Table:** 

**Figure 3:**
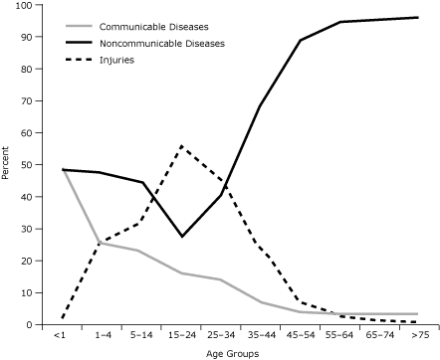
Distribution of Mortality Rates by Age Group — Palestinian Territories, West Bank, 1999-2003.

The expected pattern of epidemiologic transition is high mortality attributable to noncommunicable diseases accompanied by high mortality attributable to communicable diseases. Mortality attributable to communicable diseases was highest among children aged 4 years and younger, whereas mortality attributable to noncommunicable diseases was higher among adults (Figure 3). Mortality attributable to noncommunicable diseases increased at 35 years of age and continued to increase with age. Mortality attributable to injuries was high relative to that for communicable and noncommunicable diseases, especially among people aged 14 to 34 years.

**Table:** 

The leading cause of death for Palestinians living in the West Bank during the 5 years included in this analysis (1999-2003) was circulatory disease, followed by cancer; unintentional injury; and communicable disease, maternal and perinatal conditions, and nutritional deficiencies (Table 1). Other causes account for 26% of all deaths, and 19% of these were due to ill-defined causes. Ill-defined causes of death included senility, sudden infant death syndrome, and undefined circulatory problems or malignancies. Men had significantly higher ASMR due to circulatory diseases, cancer, unintentional injuries, and communicable diseases than did women (Table 2).

Noncommunicable diseases were common causes of death among adults (Figure 2). Therefore, we studied ASMRs for circulatory disease subgroups for adults aged 40 years or older (Table 3). Men had higher ASMRs for AMI (78.5) and chronic ischemic heart disease (20.7) than did women (34.9, 13.1). However, ASMRs for hypertension, heart failure, and cerebrovascular diseases were similar for men and women (Table 3).

Cancer accounted for 10% of total mortality in the West Bank from 1999 through 2003. We examined cancer ASMRs by site separately for men and women (Table 4). Lung cancer was the most common cause of cancer deaths among men, followed by cancers of the prostate, colon, liver and bile ducts, and stomach. Breast cancer was the most common cause of cancer deaths among women, followed by cancer of the liver and bile ducts, colon, lung, and stomach.

## Discussion

The observed mortality patterns support the hypothesis that the Palestinian Territories have been undergoing a rapid demographic and epidemiologic transition. Approximately 73% of the population is under the age of 30 years, and approximately 4% of the population is aged 65 years and older (1,6). Whereas 30 years ago major causes of death and disease were communicable diseases, maternal and perinatal conditions, and nutritional deficiencies, today we witness a rise in noncommunicable diseases without a corresponding decline in communicable diseases (13). However, this is not true for the whole population. Diseases affect different age groups within the population differently. Communicable diseases primarily affect the younger population, whereas noncommunicable diseases affect adults, especially those aged 40 years and older. The health system thus faces a double burden that is not easily manageable, especially given ongoing conflict and systemic crisis.

The observed rise in chronic disease mortality is linked to a rise in the prevalence of chronic diseases and their risk factors. Results from cross-sectional studies in the West Bank showed high prevalences of diabetes in rural (10%) and urban (12%) areas (14). A similar pattern was found for hypertension (25% in rural areas and 22% for urban areas). Furthermore, these studies have identified major risk factors for chronic diseases such as overall and central obesity (15) and hypertriglyceridemia (14). Reports from demographic health surveys also indicate a rise in the prevalence of chronic diseases. For instance, reported diabetes, hypertension, and cardiac diseases among all age groups in the West Bank have increased from 2.1%, 2.4%, and 0.6% in 2000 to 2.4%, 3.4%, and 1.4%, respectively, in 2004 (1). The prevalence among adults aged 35 years and older was 13.8% for hypertension and 10.2% for diabetes (1). These figures, however, may not reflect actual prevalence because of underreporting and underdiagnosis.

Cigarette smoking, a key risk factor associated with chronic diseases, was also prevalent in the West Bank, reaching 22% among those aged 10 years and older, and rates were much higher among men (41%) than among women (3%) (1). Smoking is prevalent in Arab countries; approximately 50% of young men aged 15 to 25 years and 10% of young women in the same age group are smokers. In 2000, the global leading cause of death from smoking was cardiovascular disease, followed by chronic obstructive pulmonary disease and lung cancer (16).

Death from AMI is higher among Palestinian men and women living in Jerusalem than among Israeli Jews (17). The difference is most likely attributable to different dietary patterns; the high prevalence of risk factors such as obesity, glucose intolerance, and diabetes in both populations; and the stress of the complex political situation and socioeconomic inequalities (17). Differences in access to adequate medical care between Palestinians and Israeli Jews living in the city are another factor (18).

The association between stress and trauma and increased chronic disease mortality was reported in Lebanon, which has been under war conditions for more than 15 years (19). Higher mortality from cardiovascular disease was reported for those exposed to human trauma, property losses, work-related problems, and displacement during war (19).

In developed countries, mortality has decreased for ischemic heart disease and cerebrovascular diseases (20,21). This decrease may be related to more accurate diagnostic methods, early treatment, better prevention, or better medical care. In general, however, chronic disease mortality is increasing in developing countries. The trend has been attributed to early onset of cardiovascular disease and changes in lifestyle (22).

Changes in lifestyle and rapid urbanization appear to be affecting the regional and Arab countries. High prevalences of obesity and hypertension and high cholesterol have been reported in several countries including Morocco (23), Egypt (24), and Iran (25). The main epidemiologic picture is characterized by rapid urbanization and changes in lifestyle and nutritional patterns (26), and the West Bank has similar characteristics. People are switching from eating traditional, healthy food to calorically dense fast food. At the same time, the level of physical activity has decreased because of modernization (27).

Longevity and increased life expectancy may also explain the observed change in mortality patterns (28). The Palestinian health care system has good experience in handling infectious diseases; people are living longer and being exposed to more risk factors for chronic disease.

According to the Ministry of Health report and based on data from the Palestinian cancer registry in the West Bank, crude cancer incidence in the Palestinian Territories in 1999 was 66.8 per 100,000, which was lower than cancer incidence in neighboring countries such as Jordan and Egypt (29). This number should be interpreted with caution, as it might underestimate cancer incidence in the West Bank. The observed high proportion of cancer mortality (10%) may support this argument. Therefore, more emphasis should be paid to early detection and increasing awareness of these diseases.

Results of this study show that mortality due to cancer among Palestinian men and women is lower than in other places in the world (30). According to WHO, countries in the region were grouped on the basis of location and mortality statistics into EmrD (subregion of Eastern Mediterranean region, which included Egypt, Iraq, Morocco, and Yemen) and EmrB (which included Bahrain, Cyprus, Iran, Jordan, Kuwait, and others) (30). The cancer ASMRs for Palestinian men (56.0) were similar to those reported for men in EmrD (54.8) and lower than those reported in EmrB (62.1), whereas the cancer ASMRs for Palestinian women (39.1) were lower than those reported for women in EmrB (49.0) and EmrD (46.1) (31).

Mortality rates attributable to unintentional injuries (which include war-related injuries) are high, especially for 2001 through 2003, which brought intensive invasions to certain areas of the West Bank and Gaza Strip. Further research into mortality from specific types of injuries within unintentional injuries is recommended.

Mortality patterns in the West Bank indicate an epidemiologic transition that needs to be addressed by health policy makers and planners. Assessed for the first time, these patterns are similar to mortality patterns in developing countries where mortality attributable to chronic diseases is increasing.

Our findings demonstrate that chronic disease in the West Bank is a public health problem. We emphasize the need to ascertain the risk factors associated with chronic diseases in the Palestinian Territories and to identify possible ways to address these risk factors so that these diseases can be reduced or prevented. Given this burden of death from chronic disease, medical and public health policies and interventions need to be reassessed, giving due attention to the rise of modern-day diseases in the area.

## Figures and Tables

**Table 1:** Cause-Specific Mortality Rates per 100,000 World Population, West Bank, Palestinian Territories, 1999-2003, a

Cause of Death	Women, n (%) n = 11,809	Men, n (%) n = 15,078	Total, n (%) N = 26,887
Communicable diseases^b^	781 (6.6)	1,125 (7.5)	1,906 (7.1)
Cancer	1,222 (10.3)	1,515 (10.0)	2,737 (10.2)
Diabetes mellitus	707 (6.0)	601 (4.0)	1,308 (4.9)
Diseases of circulatory system	5,579 (47.2)	6,466 (42.9)	12,045 (44.8)
Unintentional injuries	380 (3.2)	1,520 (10.1)	1,900 (7.1)
Other causes of death	3,140 (26.6)	3,851 (25.5)	6,991 (26.0)

a The total number of deaths registered from 1999 through 2003 was 27,065. Information on cause of death was missing for 178 deaths, leaving a total of 26,887.

b Communicable diseases include infectious and parasitic diseases, blood diseases and immune disorders, respiratory infections, perinatal conditions, nutritional deficiency, and maternal conditions.

**Table 2:** Age-Standardized Mortality Rates per 100,000 World Population per Year, West Bank, Palestinian Territories, 1999-2003

Cause of Death	Male ASMR (95% CI)	Female ASMR (95% CI)
All causes	800.9 (788.2-813.6)	583.4 (572.9-593.9)
Communicable diseases,^a^ maternal and perinatal conditions, or nutritional deficiencies	24.2 (21.0-27.4)	15.5 (13.0-17.9)
Cancer	56.0 (49.7-62.3)	39.1 (34.2-44.0)
Diabetes mellitus	24.8 (20.4-29.3)	23.1 (19.3-26.9)
Circulatory disease	227.8 (215.4-240.2)	161.5 (152.0-171.0)
Unintentional injuries	31.0 (27.5-34.5)	7.7 (6.0-9.4)

Abbreviations: ASMR, age-standardized mortality rate; CI, confidence interval.

a Communicable diseases include infectious and parasitic diseases, blood diseases and immune disorders, respiratory infections, perinatal conditions, nutritional deficiency, and maternal conditions.

**Table 3:** Age-Standardized Mortality Rates Attributable to Circulatory Diseases per 100,000 World Population per Year, West Bank Adult Population Aged ≥40 Years, Palestinian Territories, 1999-2003

Cause of Death	Male ASMR (95% CI)	Female ASMR (95% CI)
Hypertension	31.1 (26.5-35.7)	30.2 (26.1-34.3)
Acute myocardial infarction	78.5 (70.7-86.2)	34.9 (30.2-39.5)
Chronic ischemic heart disease	20.7 (16.8-24.6)	13.1 (10.3-15.9)
Heart failure	34.9 (30.6-39.6)	31.5 (27.4-35.6)
Cerebrovascular disease	41.0 (35.8-46.3)	35.2 (30.7-39.6)

Abbreviations: ASMR, age-standardized mortality rate; CI, confidence interval.

**Table 4:** Age-Standardized Mortality Rates for Cancer per 100,000 World Population per Year, West Bank, Palestinian Territories, 1999-2003

Type of Cancer	Male ASMR (95% CI)	Female ASMR (95% CI)
Brain	2.9 (2.2-3.6)	2.2 (1.6-2.7)
Breast	NA	7.1 (6.1-8.1)
Bladder	2.4 (1.7-3.0)	NA
Bronchus and lung	14.0 (12.5-15.5)	2.9 (2.3-3.5)
Colon	4.9 (4.0-5.8)	3.7 (3.0-4.5)
Laryngeal	1.3 (0.8-1.8)	NA
Liver and bile duct	3.6 (2.8-4.3)	3.7 (3.0-4.4)
Ovarian	NA	1.3 (0.9-1.8)
Pancreatic	2.0 (1.4-2.5)	1.6 (1.1-2.0)
Prostate	6.8 (5.7-7.8)	NA
Stomach	3.3 (2.5-4.0)	2.3 (1.7-2.8)
Uterine	NA	1.6 (1.1-2.1)

Abbreviations: ASMR, age-standardized mortality rate; CI, confidence interval; NA, not applicable.
