# Erratum, Vol 5: Issue 4

**Published:** 2008-12-15

Because of an editing error, a footnote incorrectly appeared in the article "Mortality Patterns in the West Bank, Palestinian Territories, 1999-2003." The footnote followed Table 3 in the article. Corrections were made to our Web site on October 16, 2008, and appear online at www.cdc.gov/pcd/issues/2008/oct/07_0184.htm. We regret any confusion or inconvenience this error may have caused.

